# Sex Specific Placental Accumulation and Behavioral Effects of Developmental Firemaster 550 Exposure in Wistar Rats

**DOI:** 10.1038/s41598-017-07216-6

**Published:** 2017-08-02

**Authors:** Kylie R. Baldwin, Allison L. Phillips, Brian Horman, Sheryl E. Arambula, Meghan E. Rebuli, Heather M. Stapleton, Heather B. Patisaul

**Affiliations:** 1 Department of Biological Sciences, North Carolina State University, Raleigh, NC 27695 USA; 2 Center for Human Health and the Environment, North Carolina State University, Raleigh, NC 27695 USA; 3 Nicholas School of the Environment, Duke University, Durham, NC 27708 USA

## Abstract

Firemaster® 550 (FM 550) is a commercial flame retardant mixture of brominated and organophosphate compounds applied to polyurethane foam used in furniture and baby products. Due to widespread human exposure, and structural similarities with known endocrine disruptors, concerns have been raised regarding possible toxicity. We previously reported evidence of sex specific behavioral effects in rats resulting from developmental exposure. The present study expands upon this prior finding by testing for a greater range of behavioral effects, and measuring the accumulation of FM 550 compounds in placental tissue. Wistar rat dams were orally exposed to FM 550 during gestation (0, 300 or 1000 µg/day; GD 9 – 18) for placental measurements or perinatally (0, 100, 300 or 1000 µg/day; GD 9 – PND 21) to assess activity and anxiety-like behaviors. Placental accumulation was dose dependent, and in some cases sex specific, with the brominated components reaching the highest levels. Behavioral changes were predominantly associated with a loss or reversal of sex differences in activity and anxiety-like behaviors. These findings demonstrate that environmental chemicals may sex-dependently accumulate in the placenta. That sex-biased exposure might translate to sex-specific adverse outcomes such as behavioral deficits is a possibility that merits further investigation.

## Introduction

Chemical flame retardants (FRs) are commonly applied to consumer products, such as furniture and electronics, to reduce flammability and delay their ignition. However, some FRs can migrate out of products over time, resulting in widespread human exposure^1^. Exposure to polybrominated diphenyl ether (PBDE) FRs has long been associated with behavioral and cognitive impairments in humans, including cognitive decrements and, possibly, heightened risk of autism spectrum disorder ASD^2–7^. As PBDEs have been phased out of use, they have rapidly been replaced by other compounds and mixtures including alternative brominated compounds and organophosphate esters (OPEs)^8^. The potential health consequences of these compounds, particularly the OPEs, are not clear but they are structurally similar to known neurotoxicants and neuroendocrine disruptors, raising concerns that exposure may adversely impact brain development. The present studies were conducted to ascertain the degree to which these replacement FRs accumulate in the placenta and impact sexually dimorphic non-reproductive behaviors.

**Figure 1:**
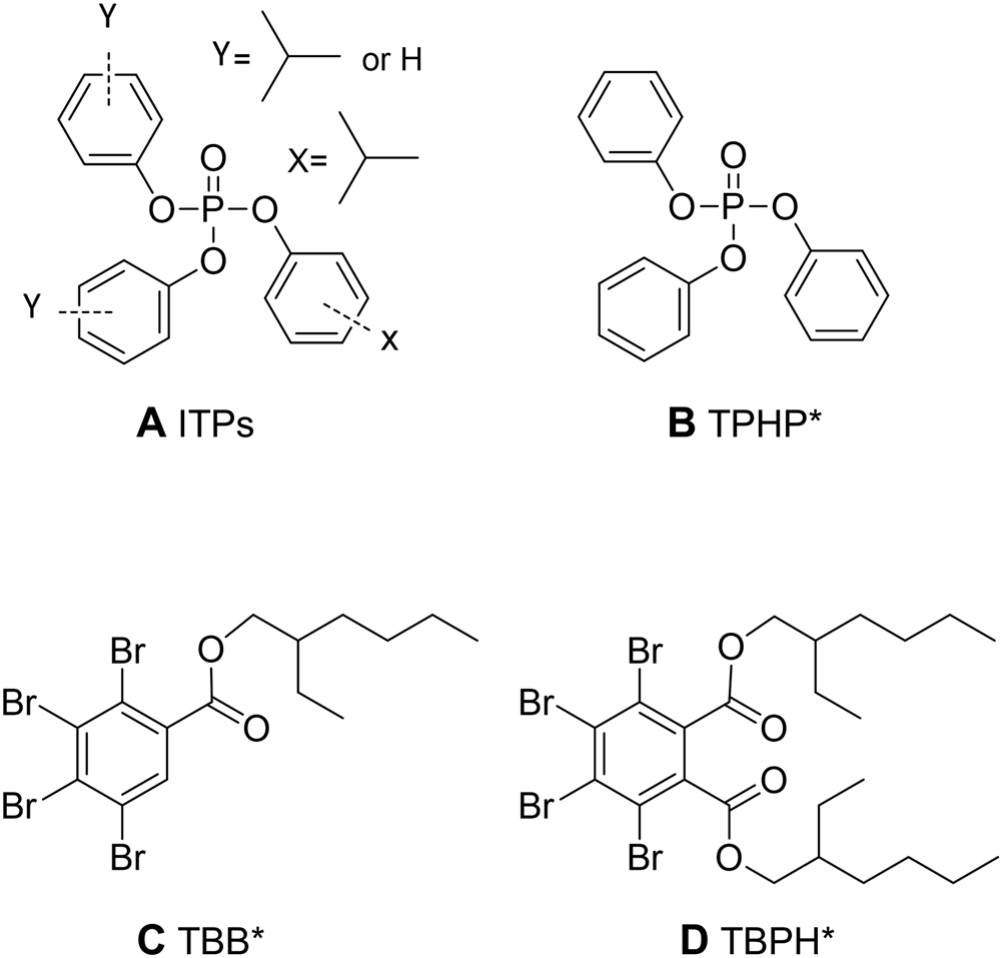
Chemical structure of the four FM 550 components. * = also known as TPP, EH-TBB, and BEH-TEBP respectively. This figure is adapted from^26^.

The alternative FR mixture known as Firemaster 550 (FM 550), is poised to be the most common flame retardant used in baby products (e.g. car seats, mattresses, nursing pillows, etc.) and residential furniture^1, 8^. FM 550 contains two brominated compounds, bis (2-ethylhexyl)-2,3,4,5-tetrabromophthalate (TBPH, also known as BEH-TEBP) and 2-ethylhexyl-2,3,4,5-tetrabromobenzoate (TBB, also known as EH-TBB), and several OPEs including triphenyl phosphate (TPP, also known as TPHP), and a mixture of isopropylated triarylphosphate isomers (ITPs) (Fig. 1)^9^. Human exposure is believed to occur primarily via inhalation, or inadvertent ingestion of indoor dust^10–14^. In the United States (US), house dust levels of FM 550 components are now equivalent to the phased out PBDEs^9^. TBB and TBPH have been measured in house dust samples from the US and globally, including the United Kingdom, Belgium, Iraq, and New Zealand at levels ranging from 3 to 15,030 ng/g^9, 15–17^ demonstrating their rapid deployment and extensive use. TBB and TBPH have also been found in breast milk, blood, hair and fingernails^18, 19^. TBB, for example, has been found in hair at levels ranging from 7.6–4540 ng/g^18^. Detection of replacement FRs, including TBB and TBPH, in atmospheric particles from the European Arctic and in marine mammals from the South China Sea underscores their persistence and their potential for long term transport and bioaccumulation^20–22^.

The OPEs found in FM550 have also been used for decades in other applications, particularly as plasticizers. TPHP in particular is a high production volume chemical (10-50 million lbs/year) and is commonly applied to polyvinyl chloride (PVC), circuit boards, hydraulic fluids, adhesives, rubbers, and other products. TPHP is also present in nail polish and urinary levels of its primary metabolite rise following application^23^, suggesting that exposure may also be occurring through personal care products. Given these exposure data and accruing evidence of endocrine disruption, both of reproductive hormones^24, 25^ and thyroid hormones^26^, there is an urgent need to investigate the toxicity and potential health effects of replacement FRs, including FM 550.

A purported no observed adverse effect level (NOAEL) has not yet been established for the full FM 550 mixture but guideline reproductive, fertility, and developmental toxicity studies on a chemical mixture (CN-2065/BZ-54) containing only TBB and TBPH established a NOAEL of 50 mg/kg/day. Testing of the full mixture is important because that is what is used commercially, and thus how humans are exposed in their homes. In a small-scale pilot study, we were the first to show that perinatal exposure to the full FM 550 mixture at levels far below (100 or 1000 µg/kg/day) this NOAEL could sex-specifically disrupt anxiety-related behaviors in adult rats^26^. Follow-up work has provided further evidence that FM 550 is a developmental neuroendocrine disruptor^26–28^. The present studies build on this collective body of work by including a more comprehensive battery of behavioral tasks to assess a greater range of non-reproductive behaviors in both sexes, and testing for behavioral outcomes at two different ages (juvenile and adult).

We have recently shown that the brominated components can be placentally transferred in rats, with detectable levels in homogenized fetal tissue from dams exposed to 1000 μg FM 550 over 10 days^29^, demonstrating the potential for fetal brain exposure. It has subsequently been shown that PBDEs sex-specifically bioaccumulate in human placenta^30^ suggesting the potential for sexually dimorphic placental FR exposures. The placenta is a significant source of hormones, monoamines and other factors which influence the developing brain^31^ suggesting that this ephemeral but neuroactive organ may be a critical but underappreciated contributor to neuroendocrine disruption and other avenues of neurotoxicity by endocrine disrupting chemicals (EDCs). Thus, the present studies also tested the hypothesis that placental accumulation of FM 550 components may be significant and sex specific.

## Methods

### Animals

Animal care, maintenance, and experimental protocols met the standards of the Animal Welfare Act and the U.S. Department of Health and Human Services “Guide for the Care and use of Laboratory Animals” and were approved by the North Carolina State University (NCSU) Institutional Animal Care and Use Committee (IACUC). A supervising veterinarian approved and monitored all procedures throughout the duration of the project. For each aim, Wistar rats were obtained from Charles River (Raleigh, NC) and bred in house as described below in humidity-and-temperature controlled rooms, each with reversed 12 h:12 h light, dark cycles at 25 °C and 45–60% average humidity at the Biological Resource Facility at NCSU. As in our prior studies^32, 33^, and in accordance with recommended practices for EDC research^26, 34–36^, all animals were housed in conditions specifically designed to minimize unintended EDC exposure including use of glass water bottles, soy-free diet (Teklad 2020, Envigo), woodchip bedding and thoroughly washed polysulfone caging. Potential litter effects were accounted for either in the experimental design (by testing only one animal per sex per group) or in the statistical analysis (by including litter as a co-variate).

### Experiment 1: Placental Accumulation of FM 550 Components

#### Dosing Prep

A concentrated ethanol solution was prepared and coded in the Stapleton lab then transferred to the Patisaul lab where oral dosing and subsequent testing was performed blinded. Each dosing solution (0 mg/ml, 20 mg/ml and 60 mg/ml) was prepared using the appropriate amount of FM550 (Great Lakes Chemical; West Lafayette, IN) diluted in 100% ethanol, stirred for 6 h, then stored in amber bottles until use.

#### Animal Husbandry and Exposure

Adult Wistar rats (n = 48 females and 26 males) were paired and monitored for the presence of a sperm plug, which was designated gestational day (GD) 0. The males were then removed and the Dams housed individually. All paired females except one were successfully impregnated and a total of 24 were used for the gestational exposure. Exposure was daily for 10 days and exclusively gestational (GD 9-18). At the time of dosing 20 µl of each dosing solution was pipetted onto ¼ of a soy-free food treat pellet (chocolate flavored AIN-76A Rodent Diet Test Tabs, Test Diet, Richmond, IN) as we have done previously^26^ resulting in three exposure groups (to which animals were randomly assigned): 0 µg FM 550 (ethanol vehicle), 300 µg FM 550 (mid), and 1,000 µg FM 550 (high). Consumption was monitored to ensure the dam ate the entire treat. Dams were not dosed by individual weight but rather by the average weight of the females before they were impregnated (300 g), producing exposures of approximately 0, 1 and 3.3 mg/kg bw per day FM 550. Additional methodological details regarding dosing and internal FM 550 exposure levels were published in a prior study using the same animals^29^.

#### Tissue Collection

Sacrifice occurred on GD 18, four hours after final dosing. All dams and fetuses were weighed and sacrificed by CO_2_ asphyxiation and rapid decapitation. For all litters, the placentas from each pup were collected and flash frozen. One placenta per sex per litter was used for the experiments reported herein. A single paw was collected from each fetus in order to determine sex via PCR as previously described^37^. Paw DNA was extracted using a DNeasy^®^ Blood & Tissue Kit (Catalog # 69504; Qiagen), according to the manufacturer’s instructions, and PCR amplification was then performed using primers for β-actin (forward, 5′-AGC CAT GTA CGT AGC CAT CC-3′; reverse, 5′-TGT GGT GGT GAA GCT GTA GC-3′) and the male specific SRY gene (forward, 5′-TAC AGC CTG AGG ACA TAT TA-3′; reverse 5′-GCA CTT TAA CCC TTC GAT TGA-3′) made by Integrated DNA Technologies (Coralville, IA). Each PCR reaction was 50 μl and contained 1 μl of extracted DNA (yielding a final concentration of 200 pg/μl), 1 μl of forward and reverse SRY (yielding a final concentration of 0.5 μM), 1 μl of forward and reverse β-actin (yielding a final concentration of 0.5 μM), 25 μl Red Taq^®^ Ready Mix™ PCR Reaction Mixture (Product # R2523, Sigma), and 20 μl of water. Cycling parameters were: 1 cycle 95 °C for 2 min, 35 cycles (95 °C 1 min, 52 °C 1 min, 72 °C 1 min), and 1 cycle 72 °C for 5 min. Finally, the PCR products were electrophoresed on a 2% agarose gel, which contained ethidium bromide for imaging, for 70 min at 50 V.

#### Placental Analysis

TPHP, TBB, and TBPH were analyzed in homogenized whole placenta (6 per sex per group) according to our previously published methods^29^ (see Supplemental Materials). ITPs were not quantified because no pure, commercially available standard for any of the ITP isomers was available. Whole placentas (approximately 0.3–0.5 g) were ground in 5 g of sodium sulfate and extracted using sonication in dichloromethane. Gravimetric analysis of a sub-sample of the extract was used to measure total lipid content of the tissue. No statistically significant differences in lipid content were observed between dosing groups or sexes, and lipid content-averaged 0.80 ± 0.26% across all placentas analyzed. Placental extracts were purified using Florisil® column chromatography, with TBB and TBPH eluted using hexane and TPHP eluted using ethyl acetate. Quantification of TBB, TBPH, and TPHP was performed by GC/MS according to our previously published methods^1, 9^ using the following standards: TBB and TBPH purchased from Wellington Laboratories and TPHP (99% pure) purchased from Sigma-Aldrich. Internal standards used for quantification were ^13^C_6_-TBB, ^13^C_6_-TBPH, and ^13^C_18_-TPHP purchased from Wellington Laboratories. Recovery standards used were ^13^C_12_-CDE 141 for TBB and TBPH and d_15_TPHP for TPHP also purchased from Wellington Laboratories. Recoveries of internal standards were 87.2 ± 8.3% for ^13^C_6_-TBB, 84.7 ± 13.9% for ^13^C_6_-TBPH, and 94.4 ± 4.2% for ^13^C_18_-TPHP. All samples were blank corrected using the average of five laboratory blanks run alongside samples. Method detection limits (MDLs) were determined using 3 times the standard deviation of the lab blanks, normalized to average placenta mass extracted. Analyte concentrations are presented on a ng/g wet weight (ng/g ww) basis. All analyses were done blinded to exposure group.

### Experiment 2: Behavioral Consequences of Developmental FM 550 Exposure

#### Animal Husbandry and Exposure

Adult Wistar rats (n = 41 females and 26 males) were obtained, maintained, and bred as described in Experiment 1. Of the 41 females paired with males, 7 failed to conceive and therefore excluded from the study. Oral exposure to the dam via food treat occurred daily from GD 9 through postnatal day (PND) 21 as described above resulting in four exposure groups: vehicle, 100 µg FM 550 (low), 300 µg FM 550 (mid), and 1,000 FM 550 (high). Assignment to exposure groups was random. Dams were dosed by an average group weight of 300 g, producing the same exposures as described in experiment one with the addition of the 100 µg FM 550 group. Litters were standardized to 10 (5:5 sex ratio whenever possible) on PND 1, with a total of 8 litters in the control group and 7 litters in each of the treatment groups. Six dams did not give birth to 10 pups, with the smallest litter containing 5 pups. These smaller litters were equally and randomly distributed across all exposure groups and thus not exposure-related. On PND 21, pups were weaned and distributed into paired (groups of 2, occasionally 3, same exposure groups) same sex littermate cages and housed in the same conditions as the Dams to ensure socialization. Over the course of the study, nine of the offspring had seizures and were excluded from the study, and 3 developed severe malocclusions requiring early euthanasia. The overall seizure rate for this study was 4%, which is higher than the reported spontaneous seizure rate for this strain of rat at 1.5%^38^. Notably, the seizure rates were highest in the low and mid dose FM 550 groups with 13.2% and 7.5% incidence of seizures, respectively but only 2.5% in the unexposed controls and 0% in the high dose FM 550 group. Incidence was too low to conduct meaningful statistical comparisons. Seizures at the low dose were split evenly between males and females while seizures were only observed in males for the control and mid dose groups.

#### Behavior Testing

Animals were assessed for general activity and anxiety-related behavior as juveniles or as adults (no animals were tested at both ages) using standard procedures (see Supplemental Materials for more detail). For all tests except the running wheel, animals were transported down a short hallway to a nearby testing room on a covered rolling cart and tested after a short acclimation period, as we have done previously^26, 33, 39^. Testing was conducted within the first 4 hours of the dark cycle, recorded, and subsequently analyzed by TopScan software (Clever Sys Inc., Reston, VA) by observers blinded to exposure group. The running wheel task was performed in the home cages and thus the animals were not transported.

Juvenile testing spanned PNDs 24–28 to ensure females would be tested prior to vaginal opening (pubertal onset)^26^. The same juvenile rats, one per sex per litter, were tested in two apparatuses: light/dark box (L/D; control: 10♀, 7♂, low: 7♀, 8♂, mid: 9♀, 8♂, high: 9♀, 6♂) and open field (OF; control: 8♀, 7♂, low: 7♀, 7♂, mid: 7♀, 6♂, high: 7♀, 5♂) using well-established methods as previously described by us and others^33, 39^. Two main endpoints were used to assess anxiety-like behavior in the L/D box: latency to enter the light side and the number of entries made into the light side within the 10 minute testing period. The light portion of the L/D box was illuminated by two 40-W clip lamps placed above the apparatus. For the juvenile OF test, testing was conducted under red light (approx. 40 lux). Anxiety-like behavior was assessed via number of center entries, duration in the center, and latency to enter the center. Activity was assessed via total distance traveled. For this measure, distance data was collected in five minute intervals, and summed over the entire 30 minute testing session (total activity). For each animal, area under the curve (AUC) over the 30-minute test was also calculated. Apparatuses were thoroughly cleaned between trials.

Adults (PND 110-120) animals were tested using L/D box (Control: 8♀, 14♂, low: 14♀, 12♂, mid: 7♀, 11♂, high: 14♀, 7♂) and then elevated plus maze (EPM; PND 185-192; control: 15♀, 19♂, low: 17♀, 19♂, mid: 13♀, 19♂, high: 21♀, 12♂) for 10 and 5 minutes, respectively (detailed in Supplementary Materials). L/D behavior was assessed as described for the juveniles using animals which had not undergone juvenile testing (two adult animals per sex per litter). All adult offspring were tested on the EPM because none had prior exposure to this apparatus as juveniles (experience confounds testing performance). EPM endpoints included number of open arm entries, duration in open arms, and duration in closed arms in order to evaluate anxiety-like behavior. EPM testing was conducted under red light. Running EPM under red light enhances exploratory behavior in a sex specific manner, with anxiety particularly decreased in females^40, 41^. During EPM testing the camera malfunctioned for two 300 µg (mid) FM 550 animals (1 male and 1 female). An additional 15 animals, 10 females and 5 males, fell off the maze and were therefore excluded from the analyses. Adult females were tested in estrus (as determined by vaginal cytology^42^ because that is the phase at which the sex difference in maze activity is most pronounced^43^. Vaginal cytology was performed two hours before behavioral testing and was started two weeks prior to behavior in order to establish that cycles were normal and ensure animals were acclimated to the process. Adult L/D box, OF, and EPM were recorded using a video camera suspended over the apparatuses. Video analysis was performed by a researcher blind to the treatment groups using TopScan software (Clever Sys Inc., Reston, VA). Video scoring was validated by hand by a second blinded observer using the program Stopwatch (courtesy of David A. Brown, Center for Behavioral Neuroscience, Emory University) as described previously^39^. Potential litter effects were controlled for in the statistical analysis (described below).

All adults (PND 160–170; control: 17♀, 21♂, low: 20♀, 19♂, mid: 17♀, 18♂, high: 22♀, 13♂) were then tested for novelty response and activity by placing a running wheel in their home cage (see Supplementary Materials for more details). VitalView software (STARR Life Sciences Corp., Oakmont, PA) was used to record wheel revolutions over the course of 63 hours for each cage starting at 14:00. Once data collection was finished, revolutions over the three days were binned into 1-hour intervals for analysis. Dark phase day 1 behavior was considered indicative of response to a novel stimulus while dark phase behavior on days 2 and 3 was considered indicative of general activity. Potential litter effects were controlled for in the statistical analysis (described below).

#### Statistical Analysis

The statistical approach was designed in accordance with published guidelines regarding low dose endocrine disrupting chemical studies with equivalent sample sizes^44^ and similarly designed studies conducted by our lab^45^. Litter effects were controlled for by either testing only one animal per sex per litter (placental deposition and juvenile L/D box and OF) or including the litter as a co-variate in the analysis (all adult behavioral analyses). Statistical analysis was performed using Graphpad Prism version 6 (La Jolla, CA). Statistical significance was set at α ≤ 0.05. The placenta data were first evaluated by two-way ANOVA with exposure and sex as main factors; Fisher’s protected Least Significant Difference (LSD) was used as the post-hoc test when appropriate. A Grubb’s outlier test was performed but none were identified. Any values that were below the method detection limit (MDL) were set as MDL/2 for statistical analyses^46^.

The behavioral data were first approached by comparing the unexposed males and females using a student’s one-tailed t-test, to check for sex differences, as we have done previously for similar studies^45^. Detection of expected sex differences (in adult EPM and L/D box) was considered validation that the testing paradigm worked properly and was sufficiently robust to detect well-established sex differences. The data for those groups were then evaluated within sex by one-way ANOVA (juvenile behavior tasks) or ANCOVA (adult behavior tasks) with exposure as the factor and litter as the co-variate. For endpoints that were not found to be sexually dimorphic, the data were analyzed similarly with sex and exposure as factors and the litter as the co-variate when appropriate (adult behavior tasks). No effect of litter was found for any endpoint except number of entries in the light side of the adult L/D box (F (3, 23) = 7.107, p ≤ 0.01). Because no litter effects were detected for any other endpoint, this single litter effect was interpreted to be spurious. Prior to assessment of main effects, outliers (no more than one per group per endpoint; 3 total from juvenile L/D box, 1 from adult EPM and 3 from adult running wheel) were identified using a Grubb’s test and removed. Removal did not affect overall results or conclusions. Significant main effects and/or interactions were followed up by one-way ANOVA with Fisher’s protected LSD as the post-hoc test. While the Fisher’s protected LSD does not provide strong family-wise error control compared to other post-hoc procedures, it was selected over more conservative options to minimize risk of Type-II error (rejecting a meaningful effect) and to be consistent with what we have done previously^45^.

Over the course of a long test, such as OF, behavior changes with experience and habituation^45, 47–49^. The first 5-min of this test are considered novel for the animal, and thus provides the most salient information about anxiety-related behaviors. As the test animal habituates, it becomes less active in the arena and typically reaches a steady state by the final 5-min of this task. As a result, activity towards the end of the task is thought to be best representative of general activity^45, 47^. Thus, juvenile OF data was analyzed using three approaches: (1) for each endpoint the data were totaled over the full 30 min testing period and compared, (2) distance traveled was analyzed by binning the data into 5-min intervals and a separate ANOVA was run for each bin, and (3) area under the curve (AUC) was calculated for distance traveled in order to evaluate overall activity over the 30-min test.

Activity wheel data was binned into 1-hour intervals over the course of the dark cycle in which animals had wheel access (8 hours on day 1 and 12 hours on days 2 and 3). For each light phase, AUC was calculated to assess overall activity during that light cycle. As with the other behaviors, expected sexually dimorphic responses between female and male controls were verified using student’s one-tailed t-test. Additionally, sex differences were compared for each 1-hour interval during the first 8 hours of activity using two-way repeated measures ANOVA and Fisher’s protected LSD. The data were then analyzed as described above using ANCOVA and Fisher’s protected LSD.

For all behavioral measures, ANOVA and ANCOVA effect size was determined by calculating an Eta squared (η^2^), effects of which are defined as small at 0.01, medium at 0.06, and large at 0.14. In cases where a main effect did not reach statistical significance but effect size was large or medium, an unprotected LSD was run to further characterize group differences. Protected and unprotected LSD effect size was calculated by Cohen’s d, effects of which are defined as small at 0.2, medium at 0.5, and large at 0.81^50^.

## Results

### Accumulation of FM 550 Components in Placenta

**Figure 2:**
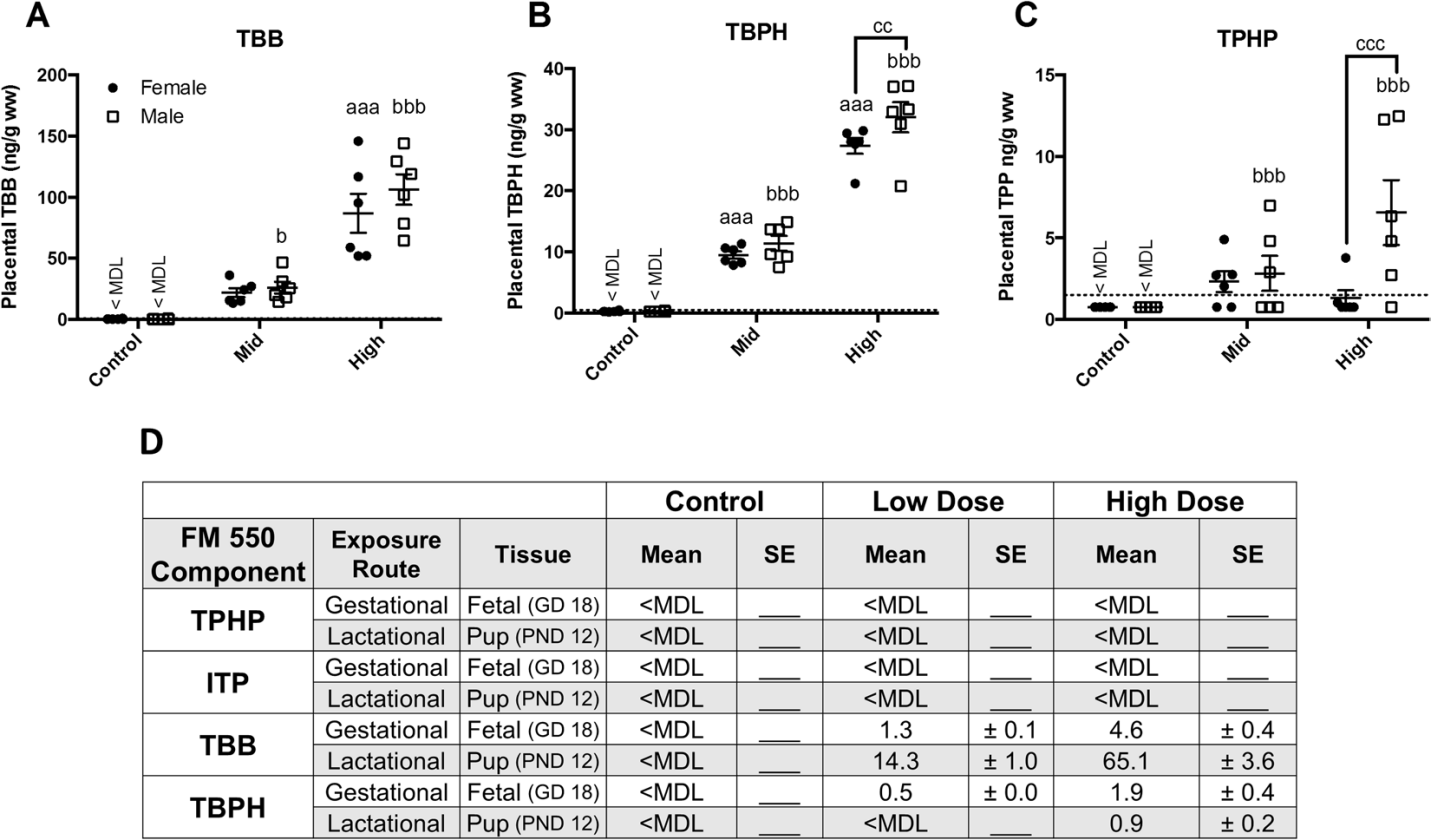
Accumulation of TBB, TBPH, and TPHP in placental tissue following gestational exposure to FM 550. Dose dependent accumulation of TBB, TBPH and TPHP (ng/g wet weight) was observed (**A**–**C**). Sex specific accumulation was statistically significant at the highest dose for TPHP and TBPH, with males having higher levels than females (**B** and **C**). The table (**D**) summarizes previously published data from our prior, related study, showing the degree to which each component was able to accumulate in fetal and pup tissues following gestational or lactation exposure, respectively (adapted from^29^). Closed circles represent females and open squares represent males. The method detection limit for TBB (0.6 ng/g wet weight), TBPH (0.5 ng/g wet weight), and TPHP (1.5 ng/g wet weight) are depicted by a dotted line. For each dose n = 6, a and b denotes statistically significant exposure effects, within each sex, while c denotes significant sex differences (^aaa^ and ^bbb^p ≤ 0.0001; ^b^p ≤ 0.05; ^cc^p ≤ 0.01; ^ccc^p ≤ 0.001). Graphs depict mean +/− SEM.

The brominated components (TBB and TBPH) accumulated in the placenta to a greater degree than TPHP, with TBB levels being the highest of the three components measured (Fig. 2). Dose dependent placental accumulation was observed for TBB and TBPH but sex-specific effects varied. TPHP also showed a dose dependent increase but only in male-associated placentas. TBB accumulation differed by exposure group (F (2, 30) = 67.59, p ≤ 0.0001; η^2^ = 0.81), but not sex and there was no significant interaction (Fig. 2A). TBB concentrations averaged 21.2 ± 3.6 ng/g ww in female-associated placentas and 25.3 ± 4.8 ng/g ww in male-associated placentas in the mid dose exposure groups, and 86.1 ± 15.9 ng/g ww in female-associated placentas and 105.5 ± 12.5 ng/g ww in male-associated placenta in the high dose exposure groups. Mean TBB concentrations in the unexposed controls were below the method detection limit (MDL) of 0.6 ng/g ww. For TBPH, a significant effect of exposure (F (2, 30) = 278.6, p ≤ 0.0001; η^2^ = 0.95) and sex (F (1, 30) = 4.54, p ≤ 0.04; η^2^ = 0.01) was observed, but there was no significant interaction (Fig. 2B). A significant effect of sex on TBPH accumulation was found in the high dose group with levels significantly higher in male placentas (p = 0.01; d = 0.96). Mean placental TBPH levels were 8.9 ± 0.5 ng/g ww for females and 10.8 ± 1.2 ng/g ww for males in the low dose exposure groups, and 26.8 ± 1.3 ng/g ww in female-associated placenta and 31.5 ± 2.4 ng/g ww in male-associated placenta in the high dose exposure groups. Average TBPH concentrations were below the MDL of 0.5 ng/g ww for both female and male placentas from the control group.

Placental TPHP levels were close to the MDL in all three groups, and each group had individuals with levels below the MDL of 1.5 ng/g ww (Fig. 2C). All of the control animals, 2/6 females and 3/6 males in the mid dose group, and 4/6 females and 1/6 males in the high dose had TPHP levels below the MDL. Two-way ANOVA revealed a significant effect of exposure (F (2, 30) = 5.3, p ≤ 0.01; η^2^ = 0.19) and sex (F (1, 30) = 5.7, p ≤ 0.02; η^2^ = 0.10), as well as a significant interaction (F (2,30) = 4.4, p ≤ 0.02; η^2^ = 0.15). A dose dependent increase in TPHP was only detected in placentas associated with male fetuses with levels in the high dose males averaging 6.5 ± 2.02 ng/g ww and levels in the high dose females averaging 1.07 ± 0.55 ng/g ww (p ≤ 0.0007; d = 1.38). These high dose averages include MDL/2 values, as even at the highest dose used, levels were < MDL for some animals. Fetal levels from this same experiment (same animals) were reported in a prior study and, summarized in Fig. 2D along with offspring levels following lactational transfer (from an accompanying study using different animals)^29^. For all compounds examined, placental levels were appreciably higher than fetal levels.

### Juvenile Behavior

**Figure 3:**
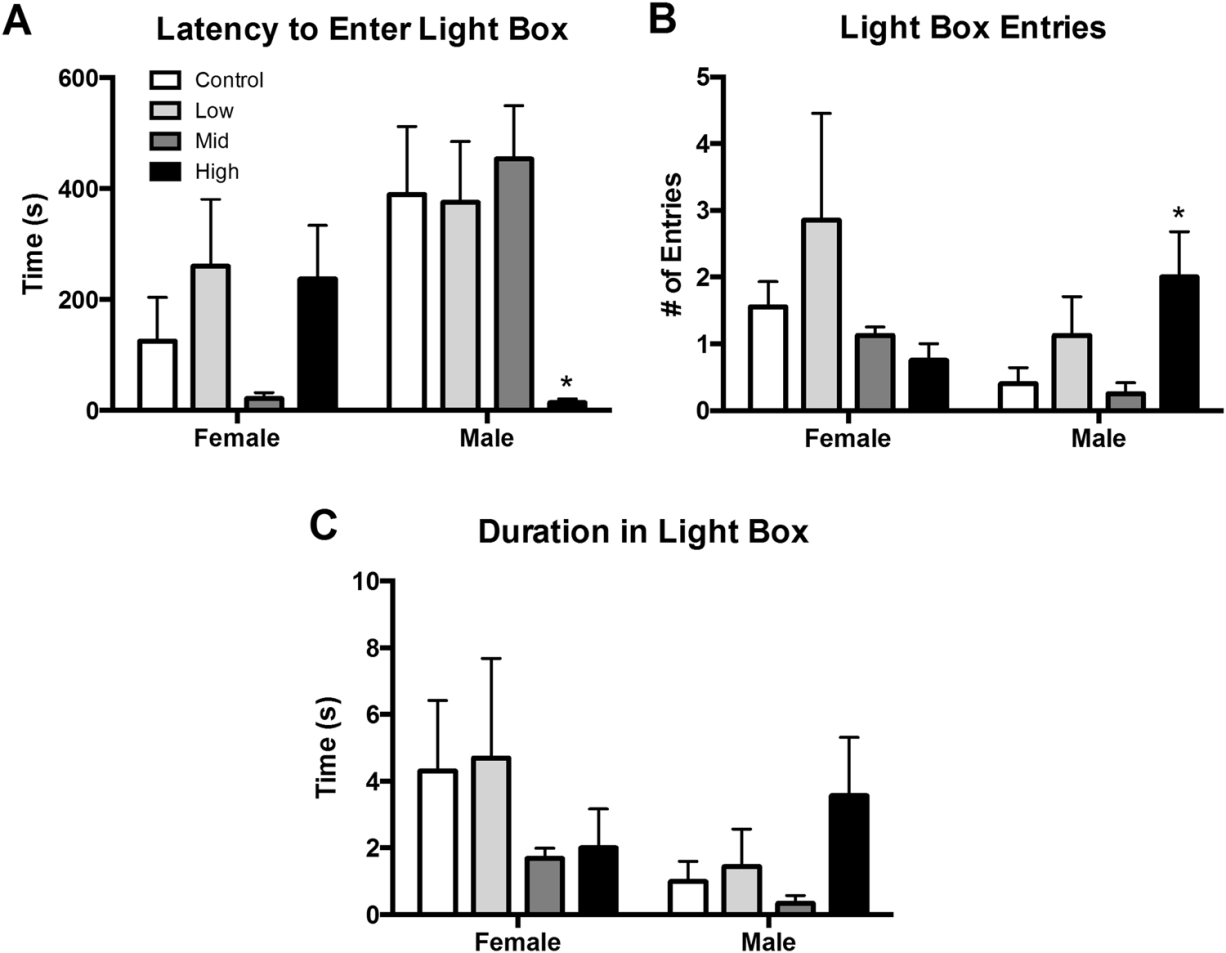
Effects of perinatal FM 550 exposure on juvenile behavior in the light dark box (L/D). No significant effect of sex was observed between control males and females for any of the L/D box measurements. In females, there was no significant effect of exposure for any of the observed endpoints. In males, latency to enter the light box was significantly reduced and light box entries were significantly increased at the highest dose (**A** and **B**), while no significant effect of exposure was observed for duration in the light box (**C**). White bars indicate control groups, light gray low dose, dark grey mid dose, and black high dose. Graphs depict mean ± SEM (*p ≤ 0.05).

Juvenile L/D box behavior was not sexually dimorphic and exposure had no main effect on females (Fig. 3). In males, however, latency to enter the light side was significantly altered by exposure (F (3, 23) = 3.092, p ≤ 0.05; η^2^ = 0.28) with high dose males taking significantly less time to enter the light box compared to unexposed controls (p ≤ 0.03; d = 0.97; Fig. 3A). Although not statistically significant, the ANOVA results were suggestive of an exposure effect on light side entries (F (3, 23) = 2.646, p = 0.07; η^2^ = 0.25; Fig. 3B). Because the effect size was large, an unprotected LSD was run to further examine potential group differences. High dose males made significantly more entries into the light box compared to control males (p ≤ 0.04; d = 0.88), with a large effect size. Time in the light side was not impacted by sex or exposure (Fig. 3C).

**Figure 4:**
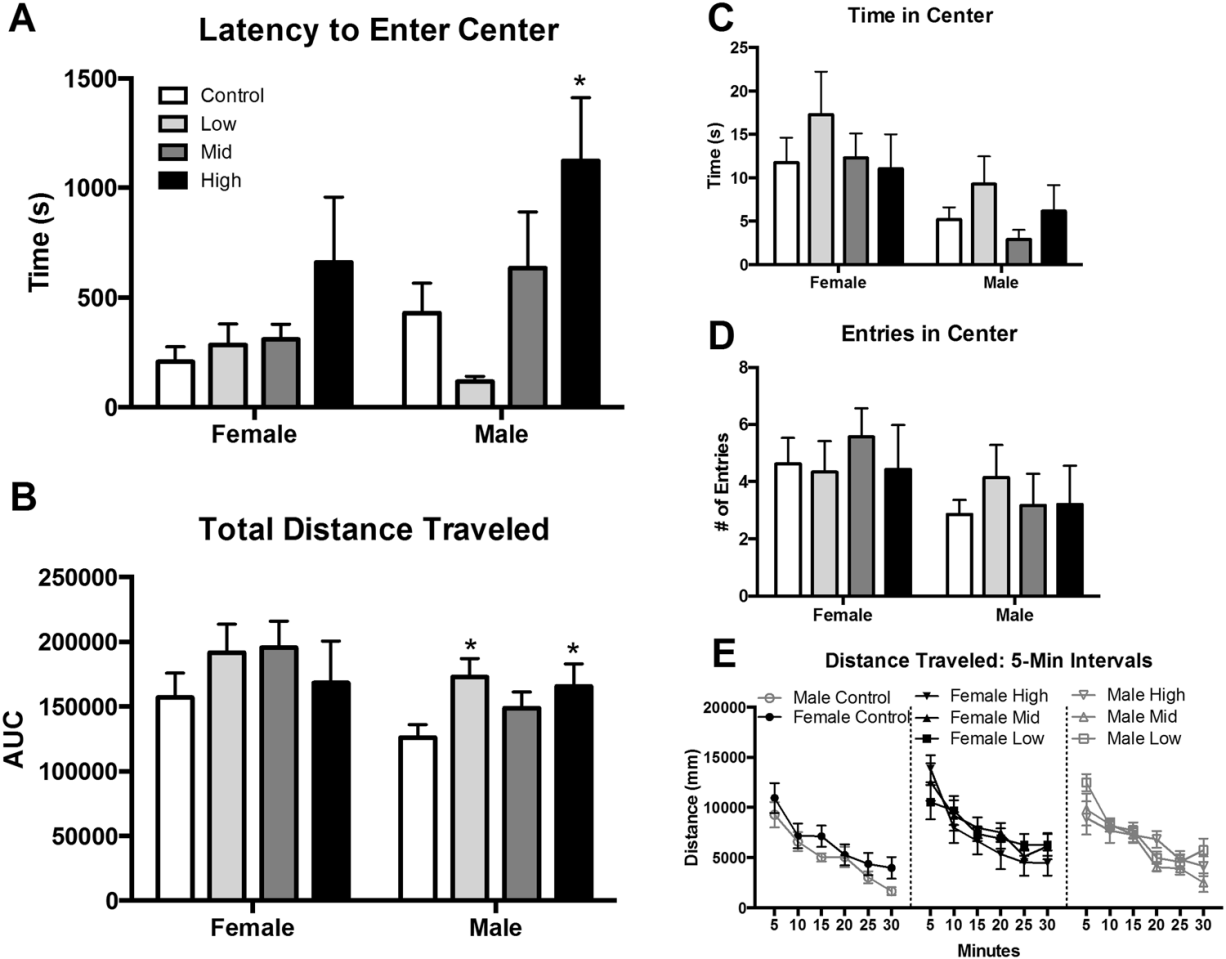
Effects of perinatal FM 550 exposure on juvenile behavior in the open field (OF). No significant effect of sex was observed between control males and females for any of the OF measurements. In females, there was no significant effect of exposure for any of the observed endpoints. In males, latency to enter the center was significantly increased for high dose males and distance traveled was significantly elevated for both low dose and high dose males (**A** and **B**). No significant effect of exposure for time in center or center entries (**C** and **D**). Distance traveled was binned into 5 minute intervals to evaluate the pattern of locomotor activity over the 30 minute test (**E**). No exposure-related effects were detected. White bars indicate control groups, light gray low dose, dark grey mid dose, and black high dose. Graphs depict mean ± SEM (*p ≤ 0.05).

Juvenile OF behavior was not sexually dimorphic and, similar to the L/D box, no effects of exposure were observed in females (Fig. 4). In males, a significant effect of exposure was identified for latency to enter the center (F (3, 20) = 4.380, p ≤ 0.02; η^2^ = 0.40), with high dose males taking longer to enter the center than controls (p ≤ 0.02; d = 1.13; Fig. 4A). Distance traveled also appeared to be impacted by exposure but the effect did not reach statistical significance (F (3, 21) = 2.667, p = 0.07; η^2^ = 0.27; Fig. 4B). Since effect size was large, an unprotected LSD was run to further evaluate effect of exposure on distance traveled. The unprotected LSD revealed increased exploration and activity in low and high dose males compared to controls (p ≤ 0.02 and p ≤ 0.05; d = 1.16 and 0.89) both of which were found to have large effect sizes. Distance traveled was also binned into 5-min intervals for comparison (Fig. 4E). As expected, the amount of activity decreased over time. For male offspring the apparent differences in distance traveled were significantly greater than controls at 15 and 30 minutes (F (3, 21) = 3.456, p ≤ 0.03; η^2^ = 0.33; F (3, 21) = 4.335, p ≤ 0.02; η^2^ = 0.38). At these two time points low dose males traveled significantly more than controls (p ≤ 0.008 and p ≤ 0.003; d = 1.29 and 1.59). No effect of exposure was found in the binned analysis for females. No effect of exposure was found for number of entries into or time spent in the center of the OF (Fig. 4C and D).

### Adult Behavior

**Figure 5:**
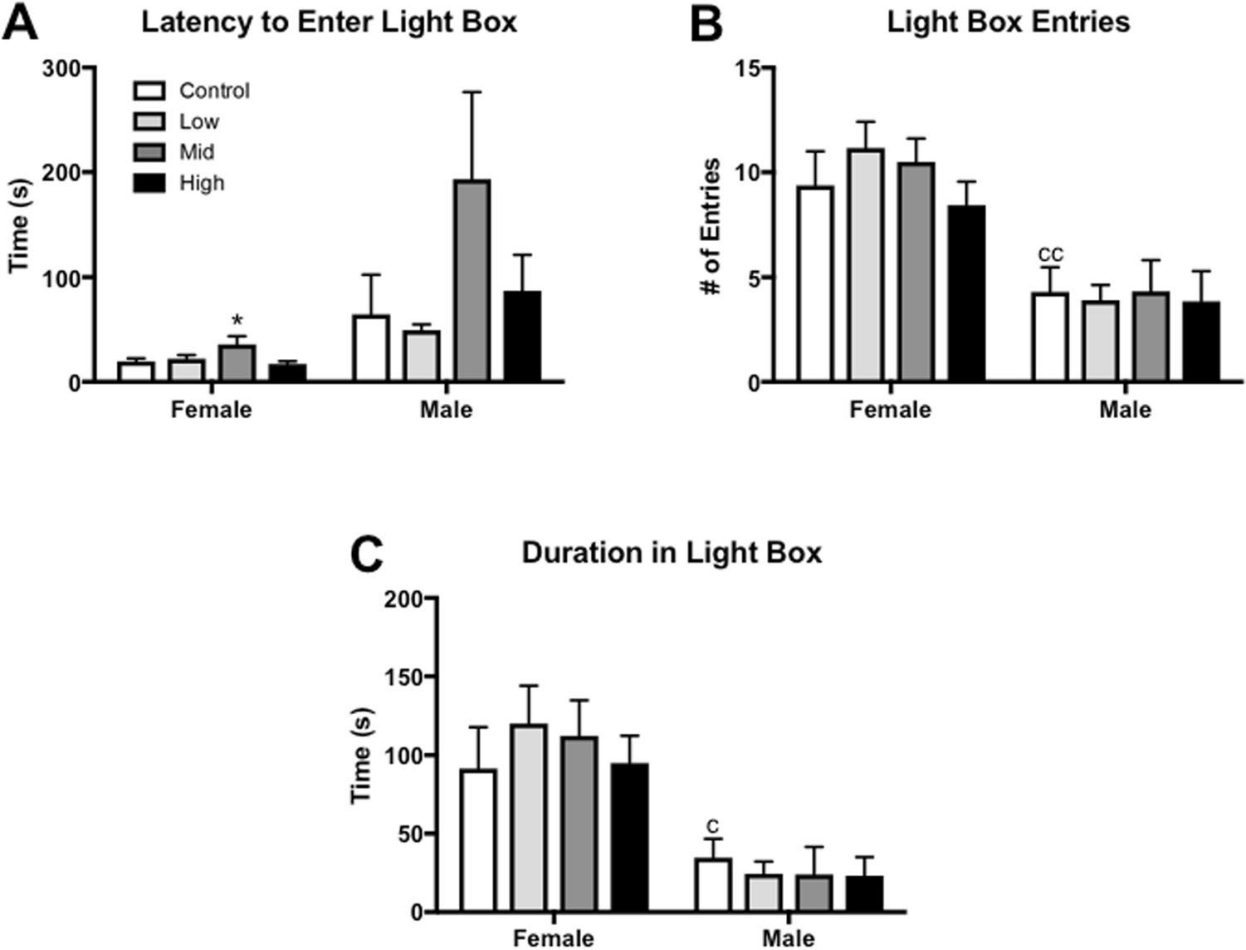
Effects of perinatal FM 550 exposure on adult behavior in the light dark box (L/D). In females, latency to enter the light box was significantly increased at the middle dose (**A**). As expected, a significant difference between male and female controls was observed for both number of entries in the light box and time spent in the light box (**B** and **C**; ^cc^p ≤ 0.01 and ^c^p ≤ 0.05). In males, no significant effect of exposure was observed for any of the L/D box measurements. White bars indicate control groups, light gray low dose, dark grey mid dose, and black high dose. Graphs depict mean ± SEM (*p ≤ 0.05).

Latency to enter the light box was evaluated, but the results should be interpreted with caution because the expected sex difference was not observed when a one-tailed t-test was run to compare the female and male control group (Fig. 5A). In females, there was a significant effect of exposure on latency to enter the light box (F (3, 35) = 3.503, p ≤ 0.03; η^2^ = 0.23). An unprotected LSD revealed that mid dose females took significantly longer than control females to enter the light box (p ≤ 0.02; d = 0.85) (Fig. 5A). As expected, adult L/D box behavior was sexually dimorphic with females making more light side entries and spending more time in the light side (p ≤ 0.01 and 0.02; d = 1.18 and 1.02; Fig. 5). No effect of exposure was observed for either endpoint or sex (Fig. 5B and C).

**Figure 6:**
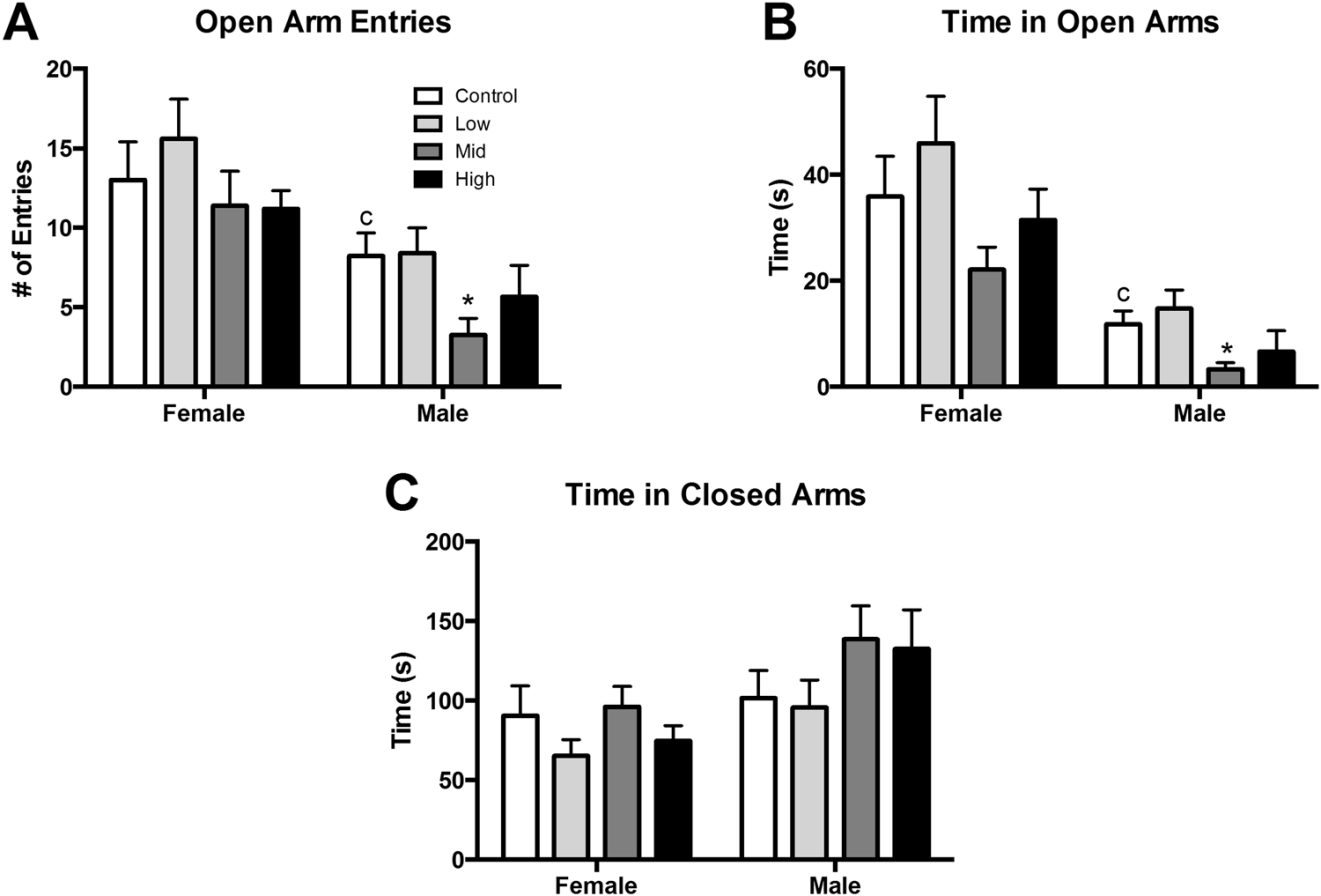
Effects of perinatal FM 550 exposure on adult behavior in the elevated plus maze (EPM). A significant difference between male and female controls was observed in both open arm entries and time in open arms (**A** and **B**; ^c^p ≤ 0.05). In females, there was no significant effect of exposure for any of the observed endpoints. In males, open arm entries and time spent in the open arms was significantly reduced at the mid dose (**A** and **B**). No significant effect of exposure was observed for time in the closed arms (**C**). White bars indicate control groups, light gray low dose, dark grey mid dose, and black high dose. Graphs depict mean ± SEM (*p ≤ 0.05).

On the EPM there was a significant effect of sex, as anticipated, for both number of entries and time spent in the open arms of the arena (p ≤ 0.05 and 0.002; d = 0.63 and 1.15; Fig. 6). No main effect of exposure was found for either endpoint in females. In males, although not quite statistically significant, an exposure effect was suggested for number of open arm entries (F (3, 58) = 2.617, p = 0.06; η^2^ = 0.12; Fig. 6A). The η^2^ of this endpoint was found to be medium, therefore an unprotected LSD was run and revealed that mid dose males entered the open arms less than controls (p ≤ 0.02; d = 0.62). A significant main effect of exposure was found for time spent in open arms (F (3, 56) = 3.23, p ≤ 0.03; η^2^ = 0.15; Fig. 6B). Mid dose males spent significantly less time in open arms than control males (p ≤ 0.03; d = 0.58). No effect of exposure was found for closed arm activity in either sex (Fig. 6C).

**Figure 7:**
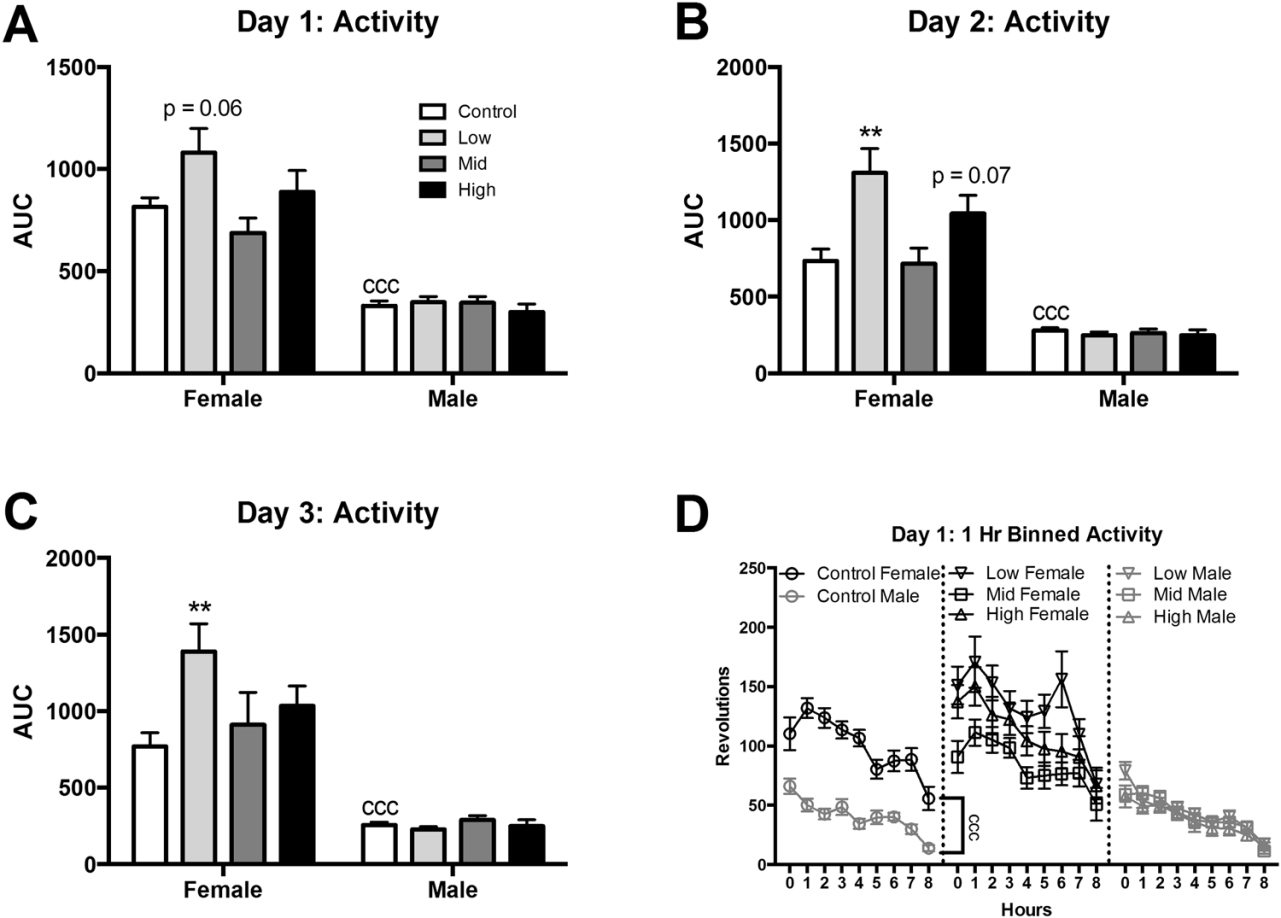
Effects of perinatal FM 550 exposure on adult behavior in activity wheels. A significant difference between male and female controls was observed for all three days of activity wheels (**A**–**C**; ^ccc^p ≤ 0.0001). In females, activity levels were significantly increased on the second and third day of activity wheels at the lowest dose (**C** and **B**). In males, there was no significant effect of exposure on any of the three days. White bars indicate control groups, light gray low dose, dark grey mid dose, and black high dose. Graphs depict mean ± SEM (**p ≤ 0.01).

All three days of running wheel testing revealed the expected large sex difference in activity^45, 51^ with females running significantly more than males (p < 0.0001; d = 3.36, 2.1, and 2.1 for days 1-3 respectively; Fig. 7). As expected, the acrophase for all animals was during the dark cycle, which is when nocturnal animals, like rats, are naturally more active^51^. No main effect of exposure on activity was found on any of the three days for males. There was however a significant effect of exposure on activity for females on all three days (F (3, 70) = 2.803, p ≤ 0.05; η^2^ = 0.10; F (3, 70) = 5.205, p ≤ 0.003; η^2^ = 0.18; F (3, 68) = 2.777, p ≤ 0.05; η^2^ = 0.11 respectively). Although not quite statistically significant, on day one low dose females were more active than control females (p = 0.06; d = 0.46; Fig. 7A). On days two and three low dose females were significantly more active compared to control females (p ≤ 0.002 and 0.008; d = 0.79 and 0.66 respectively; Fig. 7B and C). Activity wheel data was also binned into 1 hr intervals in order to examine the relationship in activity over the course of the dark cycle (Fig. 7D). Similar to the averaged data, control females were significantly more active than control males over the entire 8 hr period of the first day (F (1, 36) = 94.76, p ≤ 0.0001; η^2^ = 0.41). As expected the binned analysis showed a decreasing trend in activity over time for all groups. Activity in the light cycle was minimal, as expected, and no group differences were detected, confirming no disruption of sleep/activity patterns.

## Discussion

This study reveals, for the first time, that FM 550 components can accumulate in placental tissue, with some evidence that levels are higher in placentas associated with male fetuses. For both sexes, placental deposition did not differ by uterine position (data not shown). Placental accumulation was dose-dependent for all three compounds tested, and the magnitude of accumulation was greater for the brominated components than the organophosophate TPHP, likely due to the greater metabolism of TPHP. Disruption of normal placental function due to chemical exposure has gained interest as studies have shown that the placenta plays a significant role in fetal programming^52–55^. Our findings support the emerging hypothesis that the placenta may be a critically important target organ via which gestational exposure to FM 550 and other fire retardants could sex-specifically disrupt the fetal environment^54^. This study also extends and supports our prior report that perinatal exposure to FM 550 can affect anxiety-related behaviors in adult rats^26^. Behavioral outcomes in the present study varied to some degree across sex, age and task, but were generally indicative of heightened anxiety in males and hyperactivity in females. These results provide further evidence that perinatal exposure to FM 550 at levels below a purported NOAEL of 50 mg/kg/day, can impact neurodevelopment in a sex-specific manner.

When collectively considered with our prior study, the data reveal that the brominated components of FM 550, although capable of fetal transfer, accumulate to a greater degree in placenta^29^. Conversely, while TPHP dose-dependently accumulated in placenta, levels were low and not detectable in fetal tissues (Fig. 2). These observations are not entirely unexpected given what is currently known about the metabolism of these compounds. TPHP is rapidly metabolized^29^ but TBB is metabolized more slowly, with peak levels of its primary metabolite, 2,3,4,5-tetrabromobenzoic acid (TBBA), appearing in urine of rats 3 hrs after exposure^10^. TBPH appears to be recalcitrant to metabolism^56, 57^, at least in rats. The other organophosphate ester “component” of FM 550 was not assessed because an authentic standard for isopropylated triarylphosphate isomers (ITPs) was not commercially available, and thus not readily conducive to quantification. Here, we tested the full mixture because it is used commercially and thus how humans are exposed, but ongoing work by us and others is concurrently exploring the possible toxicity and modes of action of the individual components^58–62^.

Although the placenta plays an important role in regulating the fetal environment^54^, relatively few studies have examined the capacity for environmental contaminants, including FRs, to bioaccumulate in placental tissues, let alone if deposition can be sex specific^30, 63, 64^. The data reported herein are consistent with the recent observation that PBDE levels are higher in human placental tissues from boys than girls^65^. Rat placental levels of FM 550 components appeared to have a sex bias, with accumulation of TPHP and TBPH significantly greatest in males. By contrast, fetal levels did not appear to be sexually dimorphic but were likely too low to meaningfully test this possibility (Fig. 2D). Especially in instances where tissue levels were below or close to the limit of detection in the fetus and/or the placenta, higher exposures would be needed to provide full resolution in terms of possible sex differences in bioaccumulation, and the relationships between placental and fetal levels. Because we used whole placenta for these studies, it is not possible to determine if accumulation occurred on the maternal side, fetal side, or both. Establishing the degree to which accumulation occurs specifically within the maternal and fetal placental compartments will be the focus of subsequent studies. Additionally, information about FM 550 components in human placenta is unavailable at this time.

For the behavioral studies, the rats were exposed across gestation and also lactation because we have previously established that milk can be a significant source of exposure and thus could impact neonatal brain development^29^. Lactational transfer, particularly of the brominated components, appears to be more efficient than gestational. For example, oral administration of the same FM 550 doses as those used here and over the same length of time (10 days), produces offspring with TBB body burdens that are 200–300 times higher during lactation compared to gestation^29^ (Fig. 2D). In humans, lactational exposure also occurs^19^, with levels of TBB in breast milk higher than those of TBPH.

Behavioral outcomes were generally concordant with what we reported in our prior, exploratory study^26^, with loss or reversal of sex differences being one of the most consistently observed outcomes. In our previously published pilot study, only adults were tested and both sexes displayed evidence of heightened anxiety as adults in an elevated zero maze (a modified version of the classic EPM), while only females showed this response in the EPM. This prior study was extremely limited, however, in terms of sample size (only 2–3 dams per group). Here, in addition to sample size, we expanded the range of behavioral tasks and diversity of stimulus conditions. Importantly, we added tasks which assess general activity with the hypothesis that exposure may lead to hyperactivity. Although sample size was more than double our prior study, some results did not reach statistical significance, even though they had large effect sizes, suggesting statistical power was likely suboptimal. Consequently, the possibility that interindividual variability obscured an exposure-related signal cannot be entirely ruled out. To address this and provide greater resolution, post-hoc LSD tests were performed when effect size was determined to be large or medium.

In unexposed juveniles, no statistically significant sex differences were observed in either task, but the behaviors trended in the expected direction. This observation is consistent with a wealth of data showing that these behaviors may not become fully sexually dimorphic until after puberty^66–69^. No exposure-related effects were detected in juvenile females, but exposure-related outcomes were found in the juvenile males. Notably, they were collectively consistent with feminization. For example, in the L/D box, high dose juvenile males made more entries into the light side and latency to enter was profoundly lower compared to unexposed controls, behaviors that are typically observed in control females. This could also be interpreted as reduced anxiety, but that interpretation would conflict with the observations made in the OF, particularly greater latency to enter the center region. Hyperactivity is an alternative to the feminization hypothesis. The observation that both low dose and high dose males traveled a greater distance in the OF compared to unexposed controls, a pattern more consistent with female-typical levels of exploration, is consistent with both interpretations. It is plausible that the exposure-related L/D outcomes are also indicative of hyperactivity and increased exploratory behavior rather than a change in anxiety. Because altered exploratory behavior can confound interpretations of anxiety-related behaviors^70^, we deemed the results inconclusive in terms of possible effects on juvenile male anxiety but suggestive of hyperactivity and/or feminization. Further work using tasks which better distinguish between motivational and activity-related behaviors are needed to resolve this and establish with greater confidence the degree to which perinatal FM 550 exposure alters juvenile behaviors. Regardless, males appear to be more vulnerable.

With the exception of latency to enter the light side of the L/D box, expected sex differences were detected in all of the adult tasks, confirming that they were robust, correctly conducted, and sufficiently powered for effect sizes of this magnitude^45, 66^. As with the juveniles, L/D data was considered least valuable. Because the slight but significant increase in latency to enter the light side by mid-dose females was observed in the one measure where the controls were not sexually dimorphic, this singular effect should be viewed with caution. Additionally, adult L/D box is the only task for which a possible litter effect was identified.

Outcomes on the EPM were less ambiguous. Mid-dose males made fewer entries and spent less time in the open arms of the EPM, a classic indicator of heightened anxiety. Effects at the high dose were not statistically significant but directionally consistent. That exploration of the closed arms and running wheel behavior were unaffected by exposure in males, demonstrates that anxiety-related measures were not confounded by altered exploratory activity.

Running wheel behavior was the only measure found to be impacted by FM 550 exposure in adult females. As anticipated, profound sex differences were found in the unexposed controls throughout the test, with females more active than males. Both sexes also showed normal rhythmicity in activity with peaks just after lights off, and just before lights on suggesting that sleep patterns were not disrupted^51^. Low dose females appeared to be hyperactive for all three days of testing, including the first day when greater interest in the wheel could also be interpreted as indicative of heightened novelty seeking, but no statistically significant effects were seen at the other two doses. It is unclear as to why no consistent dose response was detected for this or any of the other tasks but, because FM 550 is a mixture, this result could be reflective of differing compensatory responses to exposure across the doses (for example, changes in hormone secretion or metabolism of the individual compounds).

Finally, that adult behavioral phenotypes differed from the juvenile ones is not unexpected given the profound neural and other changes that occur with age, including the maturation of the hypothalamic-pituitary-gonadal axis and the presence of adult-level circulating hormones^67, 68^. Many behaviors and, consequently, exposure-related behavioral phenotypes can be lost or transformed with age, including sexually dimorphic play behavior and aspects of ultrasonic vocalization. Almost nothing is known about how developmental exposure to FM 550, particularly at environmentally relevant levels, impacts the developing brain and the results herein support the need for a more comprehensive behavioral assessment including examination of cognitive and reproductive behaviors. Future studies should further increase the number of litters to boost the sample size and ensure they are sufficiently powered to test for sex-specific effects.

This study provides novel evidence that FM 550 can accumulate in placental tissues, and to a greater degree in males than females for some components. Follow-up studies will probe the mechanisms by which this sex-specific accumulation results, and the degree to which exposure might alter placental function, particularly hormone secretion. This study also provides supporting evidence that perinatal exposure heightens anxiety-related behaviors in males, and novel evidence of hyperactivity in adult females. The placenta provides chemical signals to the developing fetus that are critical for proper neurodevelopment^52, 54^. A rapidly emerging array of data is revealing that placental dysfunction as a result of environmental insult can disrupt normal brain development and behavior^31, 52^, suggesting that the placenta warrants greater attention as a potential target of toxicity and endocrine disruption by FRs and other contaminants. Linking developmental exposure with unambiguous phenotypic outcomes has yet to be fully achieved in rodent models for FM 550, but these data support the hypothesis that FM 550 may alter neurodevelopment.

## Electronic supplementary material

Supplementary Information
