# Supplementary material for: Sex Specific Placental Accumulation and Behavioral Effects of Developmental Firemaster 550 Exposure in Wistar Rats
Source: Sci Rep. 2017 Aug 2;7:7118. doi: 10.1038/s41598-017-07216-6 (PMC5540931; doi:10.1038/s41598-017-07216-6)
Supplement: Supplementary file 1 — Supplementary Information [file 41598_2017_7216_MOESM1_ESM.doc]

**Sex Specific Placental Accumulation and Behavioral Effects of Developmental Firemaster® 550 Exposure in Wistar Rats**

Kylie R. Baldwin^1^, Allison L. Phillips^3^, Brian Horman^1^, Sheryl E. Arambula^1^, Meghan E. Rebuli^1^, Heather M. Stapleton^3^, and Heather B. Patisaul^1,2^

^1^Department of Biological Sciences, North Carolina State University, Raleigh, NC 27695, USA

^2^Center for Human Health and the Environment, North Carolina State University, Raleigh, NC 27695, USA

^3^Nicholas School of the Environment, Duke University, Durham, NC 27708, USA

**Detailed Methods: Placental Analysis**

***MDL vs LOQ***

The MDLs reported herein were determined from laboratory processing blanks (n=5) by calculating 3 times the standard deviation of the blanks. LOQ values are often calculated by using a value equivalent to 10X the standard deviation of the lab blanks (or a signal to noise ratio of 10 if not detected in the lab blanks).  Therefore, MDL values represent the minimum concentration of a substance that can be measured and reported (with 99% confidence) to be greater than zero (after blank subtraction).  Thus we have chosen to use these values as our measure of quality assurance. This means that only 1% of the samples will be false positives (type I error)^1^. MDLs are a relative measure of the performance of a particular lab, method, or analyst and allow for comparison among labs and methods. MDLs are used widely in the flame retardant literature ^2-4^.

***Extraction***

TPHP, TBB, and TBPH were analyzed in whole placenta according to our previously published methods ^5^.  ITPs were not quantified in placenta tissue due to a lack of pure, commercially available standard for any of the ITP isomers.  Whole placentas (approximately 0.3 – 0.5 g) spiked with internal standards were ground in 5 g of sodium sulfate and extracted using sonication in dichloromethane.  Gravimetric analysis of a sub-sample of the extract was used to measure total lipid content of the tissue.  No statistically significant differences in lipid content were observed between dosing groups or sexes, and lipid content-averaged 0.80 ± 0.26% across all placentas analyzed.  Placental extracts were purified using Florisil® column chromatography (8 g), with TBB and TBPH eluted using hexane (24 mL) and TPHP eluted using ethyl acetate (30 mL). Extracts were concentrated under a gentle stream of nitrogen and spiked with recovery standards prior to analysis.

***Quantification***

Quantification of TBB, TBPH, and TPHP was performed by GC/MS according to our previously published methods ^6,7^. Quantification of TPHP in placental extracts was performed using an Agilent (Wilmington, DE) gas chromatograph (model 7890A) mass spectrometer (model 5975C) operated in electron impact (EI) mode. Quantification of TBB and TBPH in placental extracts was performed using an Agilent (Wilmington, DE) gas chromatograph (model 6890N) mass spectrometer (model 5975) operated in electron capture negative ionization (ECNI) mode. For all analyses, a 0.25 mm (I.D.) x 15 m fused silica capillary column coated with 5% phenyl methylpolysiloxane (J&W Scientific, 0.25 µm film thickness) and pressurized temperature vaporization (PTV) injection was used in the GC.  The inlet was set to a temperature of 80˚C for 0.3 minutes and then ramped to 300˚C at a rate of 600˚C/min to efficiently transfer samples to the head of the GC column.  The GC oven was held at 80˚C for 2 minutes, then ramped to 250˚C at 20˚C/min, then ramped to 260˚C at 1.5˚C/min, then ramped to 300˚C at 25˚C/min and held at 300˚C for 20 minutes. The transfer line temperature was held at 300˚C, and the ion source was maintained at 200˚C. TPHP was quantified by monitoring *m/z* 325 and 326; ^13^C_18_-TPHP was quantified by monitoring *m/z* 343 and 344; d_15_TPHP was quantified by monitoring *m/z* 341 and 340; TBB was quantified by monitoring *m/z* 357 and 471; ^13^C_6_-TBB was quantified by monitoring *m/z* 366 and 494; TBPH was quantified by monitoring *m/z* 463.7, 79 and 81; ^13^C_6_-TBPH was quantified by monitoring *m/z* 470 and 468; and ^13^C_12_-CDE 141 was quantified by monitoring *m/z* 388 and 390 (**Supplementary Figure 1**).

***Quality Assurance***

Recoveries of internal standards were 87.2 ± 8.3% for ^13^C_6_-TBB, 84.7 ± 13.9% for ^13^C_6_-TBPH, and 94.4 ± 4.2% for ^13^C_18_-TPHP.  All samples were blank corrected by subtracting the average mass of five laboratory blanks run alongside samples. Laboratory blank masses for TPHP, TBB, and TBPH were 1.22 ± 0.27 ng, 0.30 ± 0.09 ng, and 0.24 ± 0.11 ng, respectively.  Method detection limits (MDLs) were determined using 3 times the standard deviation of the lab blanks, normalized to average placenta mass extracted.  Analyte concentrations are presented on a ng/g wet weight (ng/g ww) basis.

| **Analyte** | **Quantifier (*m/z*)** | **Qualifier (*m/z*)** |
| --- | --- | --- |
| TPHP | 325 | 326 |
| ^13^C_18_-TPHP | 343 | 344 |
| d_15_TPHP | 341 | 340 |
| TBB | 357 | 471 |
| ^13^C_6_-TBB | 366 | 494 |
| TBPH | 463.7 | 79 & 81 |
| ^13^C_6_-TBPH | 470 | 468 |
| ^13^C_12_-CDE 141 | 388 | 390 |

**Supplementary Figure 1.** Quantifying and qualifying *m/z* for analytes and standards

**Detailed Methods: Behavior Testing**

All behavioral testing was conducted in a room at the NCSU Biological Resources Facility specifically dedicated and equipped for this purpose. All testing was done in the first four hours of the dark phase of the light cycle under red lighting (approx. 40 lux) unless otherwise indicated. All analyses were conducted by an observer blinded to exposure group and validated by hand scoring a subset of videos as we have done previously ^8,9^. Animals were quietly and quickly transported from their home room to the testing room (two doors down the hall) on a covered rolling cart to minimize distressing sights or light exposure.

***L/D Box***

The lidded L/D box testing apparatus (76 x 30 x 30 cm) was constructed of Plexiglas. The chamber is divided into two equal sides, one clear and one dark, split by a partition with an opening (10 x 10 cm) to allow passage between compartments. The light portion of the L/D box was illuminated by two 40-W (white light) clip lamps placed above the apparatus, and all activity was recorded by a camera suspended overhead. Each animal was gently placed in the dark compartment and given 10-min to explore the testing arena ^10^. Following the 5-min testing period, animals were placed back in their home cages and placed back on the rolling cart. Three L/D box testing chambers were run at a time in the same room, however each apparatus was separated from the others by an opaque divider. Males and females were never tested in the room at the same time.

***OF***

Four high walled (43 cm) blue, opaque arenas (58 cm x 58 cm) were used, each with a camera suspended overhead to record the task. All arenas were in the same room. Each animal was gently placed in the middle of the arena and given 30-min to explore the arena, during which the animals were not disturbed and there was no observer in the room. Males and females were never tested in the room at the same time. For analysis of this task the floor of the arena was digitally divided into a 3x3 square grid, 9 squares in total all of equal size, with the middle square designated as the center of the arena using CleverSys.

***EPM***

Two black Plexiglas EPM arenas were run at a time. To ensure that animals could not see or smell each other during the task, testing took place in separate rooms. This also ensured that in cases where animals fell/jumped from the maze (not atypical for juveniles or hyperactive females), the test animal could rapidly be recaptured and returned to the home cage without disturbing the other animal undergoing testing. Each apparatus consisted of 4 connected arms (15 cm wide x 50 cm long), two open (no ledges) and two enclosed (40 cm walls), that were elevated 67 cm off the floor. Each animal was gently placed in the center space of the plus facing a closed arm and given 5-min to explore the arena. Behavior was recorded by a camera suspended over each arena. Testing was done under red lighting to eliminate the additional stress of bright light.

***Activity Wheels***

Animals, one offspring/sex/litter, remained in their home cage in the room that they were housed in. A running wheel (34.3 cm in diameter) was affixed to the cage and interfaced with a computer to record revolutions per minute over the course of 2.5 days, 63 hrs. Data for a total of 23 animals was recorded at a time.

**References**

1 Resources, W. D. o. N. (1996).

2 Bradman, A. *et al.* Flame retardant exposures in California early childhood education environments. *Chemosphere* **116**, 61-66, doi:10.1016/j.chemosphere.2014.02.072 (2014).

3 Law, K. *et al.* Bioaccumulation and trophic transfer of some brominated flame retardants in a Lake Winnipeg (Canada) food web. *Environ Toxicol Chem* **25**, 2177-2186 (2006).

4 Zhu, J., Hou, Y., Feng, Y. L., Shoeib, M. & Harner, T. Identification and determination of hexachlorocyclopentadienyl-dibromocyclooctane (HCDBCO) in residential indoor air and dust: a previously unreported halogenated flame retardant in the environment. *Environ Sci Technol* **42**, 386-391 (2008).

5 Phillips, A. L. *et al.* Editor's Highlight: Transplacental and Lactational Transfer of Firemaster(R) 550 Components in Dosed Wistar Rats. *Toxicol. Sci.* **153**, 246-257, doi:10.1093/toxsci/kfw122 (2016).

6 Stapleton, H. M. *et al.* Alternate and new brominated flame retardants detected in U.S. house dust. *Environ Sci Technol* **42**, 6910-6916 (2008).

7 Stapleton, H. M. *et al.* Detection of organophosphate flame retardants in furniture foam and U.S. house dust. *Environ Sci Technol* **43**, 7490-7495 (2009).

8 Hicks, K. D. *et al.* Interaction of bisphenol A (BPA) and soy phytoestrogens on sexually dimorphic sociosexual behaviors in male and female rats. *Horm. Behav.* **84**, 121-126, doi:10.1016/j.yhbeh.2016.06.010 (2016).

9 Rebuli, M. E. *et al.* Impact of Low Dose Oral Exposure to Bisphenol A (BPA) on Juvenile and Adult Rat Exploratory and Anxiety Behavior: A CLARITY-BPA Consortium Study. *Toxicol. Sci.*, doi:10.1093/toxsci/kfv163 (2015).

10 Patisaul, H. B., Burke, K. T., Hinkle, R. E., Adewale, H. B. & Shea, D. Systemic administration of diarylpropionitrile (DPN) or phytoestrogens does not affect anxiety-related behaviors in gonadally intact male rats. *Horm. Behav.* **55**, 319-328 (2009).
